# Formulation Development and Improved Stability of a Combination Measles and Rubella Live-Viral Vaccine Dried for Use in the Nanopatch^TM^ Microneedle Delivery System

**DOI:** 10.1080/21645515.2021.1887692

**Published:** 2021-05-06

**Authors:** Ying Wan, Vineet Gupta, Christopher Bird, Swathi R. Pullagurla, Paul Fahey, Angus Forster, David B. Volkin, Sangeeta B. Joshi

**Affiliations:** a Department of Pharmaceutical Chemistry, Vaccine Analytics and Formulation Center, University of Kansas, Lawrence, KS, USA; b Vaxxas Pty Ltd, Translational Research Institute, Brisbane, QLD, Australia

**Keywords:** Measles, rubella, virus, vaccine, formulation, stability, delivery, microneedle, nanopatch™, microarray patch

## Abstract

Measles (Me) and rubella (Ru) viral diseases are targeted for elimination by ensuring a high level of vaccination coverage worldwide. Less costly, more convenient MeRu vaccine delivery systems should improve global vaccine coverage, especially in low – and middle – income countries (LMICs). In this work, we examine formulating a live, attenuated Me and Ru combination viral vaccine with Nanopatch™, a solid polymer micro-projection array for intradermal delivery. First, high throughput, qPCR-based viral infectivity and genome assays were established to enable formulation development to stabilize Me and Ru in a scaled-down, custom-built evaporative drying system to mimic the Nanopatch™ vaccine coating process. Second, excipient screening and optimization studies identified virus stabilizers for use during the drying process and upon storage in the dried state. Finally, a series of real-time and accelerated stability studies identified eight candidate formulations that met a target thermal stability criterion for live vaccines (<1 log_10_ loss after 1 week storage at 37°C). Compared to −80°C control samples, the top candidate formulations resulted in minimal viral infectivity titer losses after storage at 2–8°C for 6 months (i.e., <0.1 log_10_ for Me, and ~0.4 log_10_ for Ru). After storage at 25°C over 6 months, ~0.3–0.5 and ~1.0–1.4 log_10_ titer losses were observed for Me and Ru, respectively, enabling the rank-ordering of the stability of candidate formulations. These results are discussed in the context of future formulation challenges for developing microneedle-based dosage forms containing stabilized live, attenuated viral vaccines for use in LMICs.

## Introduction

Measles (Me) and rubella (Ru) are enveloped, single-stranded viruses with negative and positive polarity, respectively.^1,2^ Me virus infection is highly contagious and can lead to life-threatening complications. Ru virus infection is usually milder, but infection during pregnancy can cause miscarriage, fetal deaths, or congenital defects. The diseases resulting from infection by Me and Ru are preventable by vaccination.^3,4^ Live, attenuated Me and Ru viral vaccines were developed and commercially introduced in the 1960s at Merck & Co. under the leadership of Dr. Maurice Hilleman. Me and Ru containing vaccines are currently in widespread use, either individually, in combination as a divalent vaccine, or as components of a trivalent formulation (with a live, attenuated mumps vaccine, M-M-R®II and PRIORIX®) as well as a quadrivalent formulation (with live, attenuated mumps and varicella vaccine, ProQuad®).^5^ Together, Me and Ru containing vaccines have greatly reduced the infection incidence, for example, the M-M-R®II vaccine is >90% efficacious for each of the three targeted diseases with a 2-dose vaccination regime.^3–6^ Global measles vaccinations between 2000 and 2015 have been estimated to have led to the prevention of >20 million deaths worldwide.^5^

Despite the availability of such effective Me Ru containing vaccines, progress toward prevention and elimination of Me and Ru worldwide has been slower than expected, especially in the low – and middle-income countries (LMICs). Under the Global Vaccine Action Plan, Me was originally targeted for elimination in at least five of the six WHO regions by 2020, and to meet this goal, high levels of population immunity was needed to be achieved by providing high vaccination coverage (e.g., > 90% with 2-doses).^7^ This goal is especially challenging for LMICs with limited health-care infrastructure.^3^ One strategy to improve vaccine coverage is to develop more cost-effective and more convenient to use vaccination approaches.^8^ Currently, commercially available Me and Ru containing vaccines are lyophilized formulations consisting of two separate vials including the freeze-dried vaccine and the diluent for reconstitution. Thus, there is a requirement of withdrawing the reconstitution diluent into a needle and syringe, changing the needle, adding to and reconstituting the freeze-dried vaccine, withdrawing again, and then administering subcutaneously (SC) within 8 h after reconstitution (at 2–8°C).^5,9–12^ Newly emerging microarray patch (MAP) technologies have shown promise by not only greatly simplifying such vaccine administration procedures, but also to potentially enable vaccine dose-sparing by delivery via the more immunogenic intradermal route.^13–32^ MAPs consist of thousands of tiny projections with dried vaccine coated onto these projections. When applied to the skin, the patches directly deposit the vaccine into the epidermal and dermal layers to facilitate an effective immune response. By eliminating the need for needles/syringes, MAP formulations of Me and Ru vaccine targeted for use in LMICs would not only promote more convenient administration (by minimally trained healthcare worker or self-administered), but also reduced logistical costs in transport (due to small size), and potentially increased stability (during storage, temperature excursions, and/or from losses during manufacturing). Together, these advantages can potentially enable increased vaccination coverage in LMICs through the use of microneedle patch formulations and help to achieve the goal of Me and Ru elimination.^33,34,35^

Among the various MAP technologies evaluated with numerous different vaccine antigens, Nanopatch™ microprojection vaccine delivery technology has shown encouraging preclinical results for several vaccines^14,17,18,23,24,26,29,30,36^ as well as in early clinical trials.^13,15,16^ The Nanopatch™ delivery reproducibly targets a vaccine to thousands of antigen-presenting cells in both the epidermal and dermal layers of the skin.^37,38^ The combination of targeted vaccine delivery together with localized inflammation resulting from localized cell death may lead to improved immune responses over conventional needle-based intradermal delivery.^17,24^ Vaxxas Pty Ltd Nanopatch™ technology is a liquid crystal polymer (LCP)–based material, which is of low cost and is scalable for commercial vaccine production volumes.

Recent work in our laboratories reported the development of a scaled-down, laboratory model of the Nanopatch™ microneedle coating/drying process, and its implementation for screening pharmaceutical excipients and developing stabilizing dried formulations of a trivalent inactivated polio vaccine (IPV) for use with the Nanopatch™ delivery.^31^ In this work, we evaluated whether the live attenuated Me and Ru viral vaccines, which are known to be inherently less stable than IPV, could be stabilized and formulated for the same purpose. The two main goals of this work were to (1) develop analytical methodologies to determine the yield and stability of two live, attenuated viruses after being reconstituted from the Nanopatch™, and (2) develop candidate MeRu formulations with minimized *in vitro* potency losses during drying and storage in a dried state. In this study, we used both viral infectivity qPCR as well as viral genome qPCR methods to monitor virus stability and yield, respectively. We then performed a series of formulation screening experiments and identified and rank-ordered several candidate formulations of a combination live, attenuated MeRu vaccine that are not only stable under laboratory conditions that mimic the vaccine coating and drying process of Nanopatch™, but also during subsequent vaccine storage in the dried state at various temperatures.

## Materials and Methods

### Materials

Measles bulk (Edmonston-Zagreb Strain, Clarified Virus Pool No. 066M626084), rubella bulk (Wistar 27/3 Strain, Clarified Virus Pool No. 068R508026), and anti-rubella serum (equine origin), were provided by Serum Institute of India Pvt. Ltd. (SIIPL), India, and were stored at −80°C (Frozen bulk). The concentration of each virus bulk was 10^5.32^ CCID_50_/0.5 mL in a minimum essential medium (MEM), pH 7.2 with various excipients added. An in-house developed 96-well microplate drying rig was provided by Vaxxas Pty Ltd, Australia. Six millimeter diameter liquid crystal polymer (LCP) discs were provided by Cyrus Technology Pvt. Ltd., Singapore. Taqman™ Fast Virus 1-Step master mix and TaqPath™ 1-Step multiplex Master Mix TaqMan® were purchased from Applied Biosystems. Glutamic acid and lactose were obtained from Fluka. Human serum albumin (HSA) was purchased from Octapharma. Methionine was procured from MP biomedicals. Urea was purchased from Promega. Pluronic F-68 (PF68), dextran sulfate, and PEG-3350 were obtained from Spectrum Chemicals. Dithiothreitol (DTT), polysorbate 20 and 80 (PS20 and PS80), triton X-100 were obtained from ThermoFisher. Sucrose, trehalose, and mannitol were purchased from Pfanstiehl. Sulfobutyl ether beta-cyclodextrin (Captisol) was obtained from Ligand Technology. Sodium hyaluronate was obtained from Acros. All other excipients or reagents were purchased from Sigma-Aldrich.

### Methods

#### Preparation of Excipient Stock Solutions and Virus Formulations

Concentrated (2X) stock solutions of excipients were prepared in 10 mM phosphate buffer (PB), and the pH was then adjusted to targeted pH using HCl or NaOH. The solution was sterile filtered through 0.22 µm polyvinylidene difluoride (PVDF) membrane. Me and Ru bulks were mixed at 1:1 ratio to make MeRu combination bulk which was first diluted 10 fold in 10 mM PB buffer and then mixed with equal volume of 2X excipient stock solution or base buffer alone (PB) (10^4.02^ CCID_50_/mL for each virus in solution prior to drying). The LCP discs were used as surrogate for LCP-based microprojections to enable much faster experimental throughput at a laboratory scale. 10 μL of each formulated MeRu solution was then dispensed onto the center of each LCP disc in a 96-well plate and dried under N_2_ flow for 17–19 min under 14 L/min N_2_ flow in a custom drying rig at room temperature with 180°C plate rotation midway in the drying process to ensure uniform drying. The plates were then sealed with thermo-stable adhesive film and stored with desiccant (containing ~10^2.02^CCID_50_/disc of each virus). For a sample to be considered sufficiently dried, qualitative visual assessments and a “scratch test” were used. For the former, the dried formulation should form a solid thin layer (~2 mm in diameter) at the center of the LCP disc. For the latter, the dried formulation should have a solid, firm behavior when scratched by a pipette tip. After drying and storage, to reconstitute the virus, 100 µL of ice-cold reconstitution buffer (MEM containing 0.3% BSA, pH 7.2, sterile filtered) was added to each disc in the 96-well plate. The plate was then shaken at 4°C for 30 min at 300 rpm, followed by manual mixing. The freshly reconstituted samples were immediately subjected to analytical testing without any additional storage.

#### Measles and Rubella Viral Infectivity by RT-qPCR Assays

Previously reported methods for Me and Ru virus titer quantitation were adapted for determining both Me and Ru titers in this work by infecting a single cell type (Vero cells), followed by viral mRNA quantitation without the need of viral RNA extraction.^39–42^ The assay was performed by infecting 100 µL of Vero cell suspension (4 x 10^5^ cells/mL) in 96-well plates with 50 µL of serially diluted MeRu viral reference or reconstituted dried on-disc virus sample, followed by 48–50 h incubation at 37°C. MeRu frozen bulk was included in each assay as a reference, and the results were expressed as CCID_50_/mL. The infected cells were rinsed by PBS and lysed by freeze-thaw in presence of 0.45% triton X-100. The lysate was diluted 1:20 in ultrapure nuclease-free water, and one step RT-qPCR was performed using 12 µL of cell lysate in a total of 20 µL reaction mix, for amplification of the Me N gene and Ru E1 5ʹUTR cDNA sequence. mRNA produced during replication was quantitated using QuantStudio™ 7 Flex Real-Time PCR System (Applied Biosystems, USA).

For simplex RT-qPCR assays, reaction mixtures contained 1X Taqman™ Fast Virus 1-Step master mix, 900 nM of forward and reverse primers for either Me or Ru virus, and 250 nM corresponding probe labeled with fluorescent molecule FAM. For duplex RT-qPCR assay, reaction mixture contains 1X TaqPath™ 1-Step multiplex Master Mix, 450 nM of forward and reverse primers for each of Me and Ru viruses, and 125 nM probes that were differently labeled with fluorescent molecules (FAM for Me, and VIC for Ru). The primer and probes sequences are listed in Supplemental Table S1. The RT-qPCR cycling conditions consisted of reverse transcription at 52°C for 30 min, RT inactivation/initial denaturation at 95°C for 10 min, and 45 cycles of denaturation at 95°C for 15 s, annealing/extending at 60°C for 1 min.

#### Measles and Rubella Viral Infectivity by Cell Culture Infectious Dose 50% Assays (CCID_50_)

CCID_50_ assays for measles and rubella were performed on Vero and RK-13 cells, respectively, according to procedures adapted from WHO guidelines.^43^ Briefly, 100 µL of Vero (for Me, with anti-rubella serum) or RK-13 (for Ru) cell suspensions (2 x 10^5^ cells/mL) in 96-well plates were infected with 100 µL of serially diluted MeRu viral reference or reconstituted dried on-disc virus sample, followed by 10 days of incubation at 35°C, 5% CO_2_ (for Vero cells) or 32°C, 2% CO_2_ (for RK-13 cells). MeRu frozen bulks were included in each assay as a reference, and the results were expressed as CCID_50_/mL. After end of the incubation period, assay plates were examined under a microscope to count the number of wells exhibiting cytopathic effect (CPE). The number of CPE positive wells was then converted to CCID_50_ titer using the Spearman-Karber method. A pre-filled Excel sheet for the CCID_50_ calculation was downloaded courtesy of Marco Binder, Heidelberg (http://www.molecular-virology.uni-hd.de, “downloads” section) and was used for titer calculation.^44^

#### Measles and Rubella Viral Particle Concentration by Genomic RT-qPCR Assays

Measles and rubella viral RNAs were isolated from 140 µL of reconstituted dried on-disc MeRu viruses, using the QIAamp viral RNA mini kit (Qiagen, Hilden, Germany) according to the manufacturer’s protocols. Purified RNA was eluted into 30 µL of elution buffer and were immediately subjected to analytical testing without any additional storage. Genomic RT-qPCR assay shared the same primers, probes, and PCR cycling conditions with the infectivity RT-qPCR assay (as described above). As the absolute genome copy numbers of Me and Ru were not determined, the results were expressed as percentage of frozen bulk values (day 0 control).

#### MeRu Viral Yields and Stability Evaluations

To evaluate MeRu vaccine yields and stability following formulation, drying, and storage in the solid state, *in vitro* potency values of reconstituted dried on-disc MeRu vaccine samples (or non-dried liquid controls) were determined using the infectivity RT-qPCR or CCID_50_ assays as described above. MeRu vaccine stability was evaluated as percent potency, titer (potency value), or titer loss. A liquid MeRu bulk vaccine sample stored at – 80°C (frozen bulk), was included in each assay as a control and was considered to have 100% *in vitro* potency. MeRu vaccine *in vitro* potency values were measured at each of the following four stages: Stage 1 – Freshly thawed MeRu bulk stored at – 80°C (frozen bulk); Stage 2 – Freshly formulated MeRu vaccine in liquid state (non-dried liquid); Stage 3 – Freshly dried onto the LCP-disc MeRu vaccine sample (freshly dried on-disc); and Stage 4 – Dried on-disc MeRu vaccine sample stored over time at various temperatures (stored dried on-disc),

Then, four different *in vitro* potency loss values between different stages were calculated as follows: (1) Loss during formulation = Potency (frozen bulk) – potency (non-dried liquid); (2) Loss during drying = Potency (non-dried liquid) – potency (freshly dried on-disc); (3) Loss during storage = Potency (Freshly dried on-disc) – potency (stored dried on-disc); and (4) Total potency loss = Potency (frozen bulk) – potency (stored dried on-disc). Errors for vaccine *in vitro* potency losses were calculated by the propagation of error method using following equation: SE(C) =$\sqrt{SE{\left(A\right)}^{2}+SE{\left(B\right)}^{2}}$. SE(C) denotes propagated error of calculated potency loss value C, and SE(A) and SE(B) denote standard deviation of measured potency values A and B.

## Results

### Establishing high-throughput viral titer quantitation assays and virus stability screening conditions for formulation development

As a first step to initiate formulation development with the live, attenuated MeRu viruses for use in the Nanopatch™ delivery system, we required a high-throughput viral infectivity assay to monitor virus stability. To this end, we adapted and optimized previously described infectivity RT-qPCR methods for live viral vaccines including Me and Ru.^39–42,45–49^ The optimized RT-qPCR method used a single cell type (Vero cells) for Me and Ru viral infections, eliminated the need for viral RNA purification, and allowed for quantitation of viral titers after only 48 h. post infection (Supplemental Figure S1). The throughput of the infectivity RT-qPCR assay was then doubled by demonstrating similar results in a duplex vs. simplex assay format allowing for measuring viral titers of Me and Ru simultaneously in one assay.

**Figure 1.**
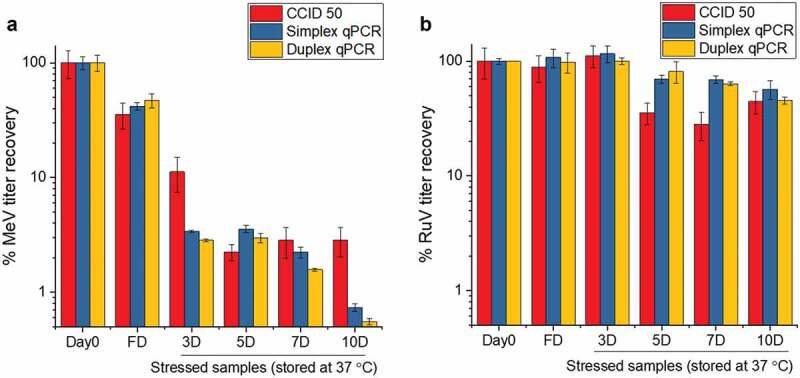
Me and Ru viral titer stability profile as measured by a high-throughput infectivity RT-qPCR and CCID50 assays. Comparison of CCID_50_, simplex and duplex infectivity RT-qPCR assays for monitoring the stability of Me (A) and Ru (B). The two viruses were combined and diluted 20X with 10 mM phosphate buffer, pH 7.2 to a final target titer of 10^4.02^ CCID_50_/mL for each virus prior to drying. Ten µL of samples were dried on each disc (10^2.02^ CCID_50_/disc for each virus), and then reconstituted either immediately after drying (fresh dried, FD), or after storage for up to 10 days at 37°C in the dried state. Me and Ru infectivity titers were measured by either CCID_50_, simplex, and duplex infectivity RT-qPCR assays, and samples were compared to a frozen liquid control stored at −80°C (percent viral titer). Error bars represent one standard deviation of quadruplicate experiments

To confirm Me and Ru stability results from the RT-qPCR assay correlated with the standard QC assay for MeRu *in vitro* potency (CCID_50_ assay), both Me and Ru were combined in a phosphate buffer (pH 7.2), air-dried onto discs, and stored at 37°C for up to 10 days. As shown in Figure 1, the remaining infectious viral titer content dropped to <5% and to <75% over 10 days, for Me and Ru, respectively. The simplex, duplex RT-qPCR, and CCID50 assays were observed to be in overall agreement to monitor virus stability (Figure 1), and a good correlation between the assays was demonstrated by the calculated Pearson coefficient r values of > 0.98 for Me and > 0.8 for Ru which indicate high positive correlations (Supplemental Figure S1).^50^ In addition, the total viral recovery for both Me and Ru close to 100% by genomic RT-qPCR, and similar stability results were observed for both Me and Ru virus after being dried on LCP discs and stored for 2 weeks at 25°C (data not shown).

**Figure 2.**
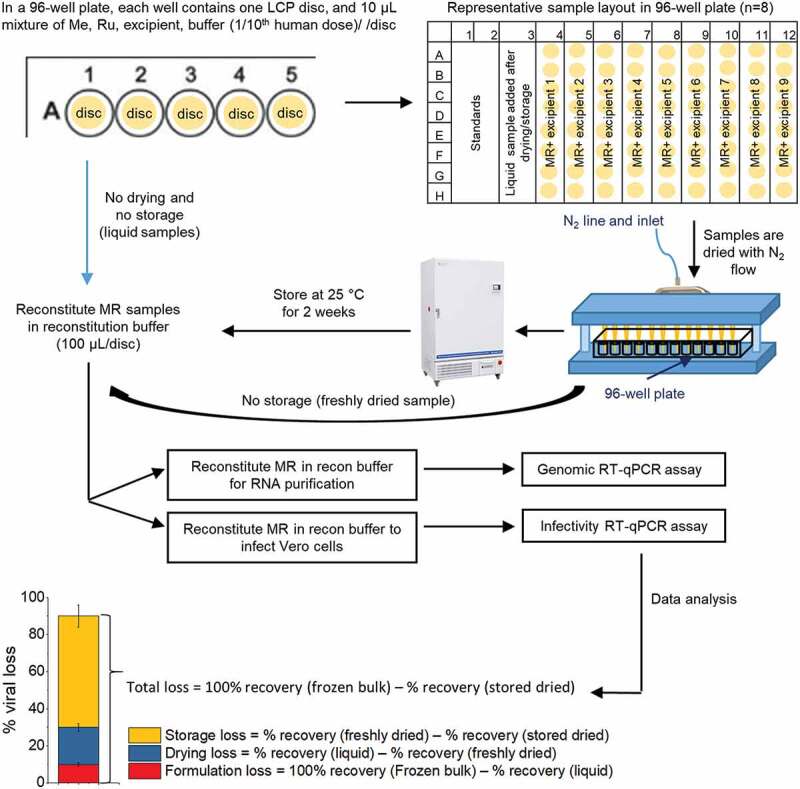
Overview of formulation experiments to assess the stability of Me and Ru (MeRu) viruses using a scaled-down lab model of Nanopatch^TM^ coating/drying process. Flowchart shows the preparation of MeRu samples in various formulations at targeted titer of 10^2.02^ CCID_50_/disc for each virus (1/10 human dose), loading of samples into 96-well plates with LCP discs, drying by nitrogen, incubation of dried samples under various storage conditions, reconstitution of samples, and then determination of viral titers and genomes by infectivity and genomic RT-qPCR assays. The bottom panel shows the data analysis procedure to determine % losses of viral titers and viral particles during formulation, drying and storage. The values of each sample were normalized to frozen bulk stored at −80°C (100%)

These storage conditions provided a good “stability window” to screen for stabilizers of Me and Ru viruses in the dried state. After drying and storage (25°C, 2 weeks in a phosphate buffer, pH 7.2), Me lost over one log_10_ of titer, and therefore the ability of various additives to improve Me titer retention can be demonstrated beyond assay variability (data not shown). At the same time, the effect of these additives on Ru, the more thermally stable virus in the combination vaccine, can also be monitored. The ability of various classes and types of pharmaceutical excipients to stabilize both MeRu in a combination vaccine was determined using the experimental outline shown in Figure 2. Briefly, Me and Ru bulks were added to various excipient samples, dried onto LCP discs, stored at 25°C for two weeks, rehydrated, and then assayed by both infectivity and genomic RT-qPCR assays. As shown in the bottom panel of Figure 2, a freshly thawed frozen liquid bulk stock solution of each virus was run as a benchmark for 100% recovery of viral titer. The Me and Ru viral titers were then measured for each sample, and results were normalized to the frozen bulk control sample (see Methods).

### Excipient screening experiments to identify potential stabilizers of MeRu during drying and storage in the dried state

**Figure 3.**
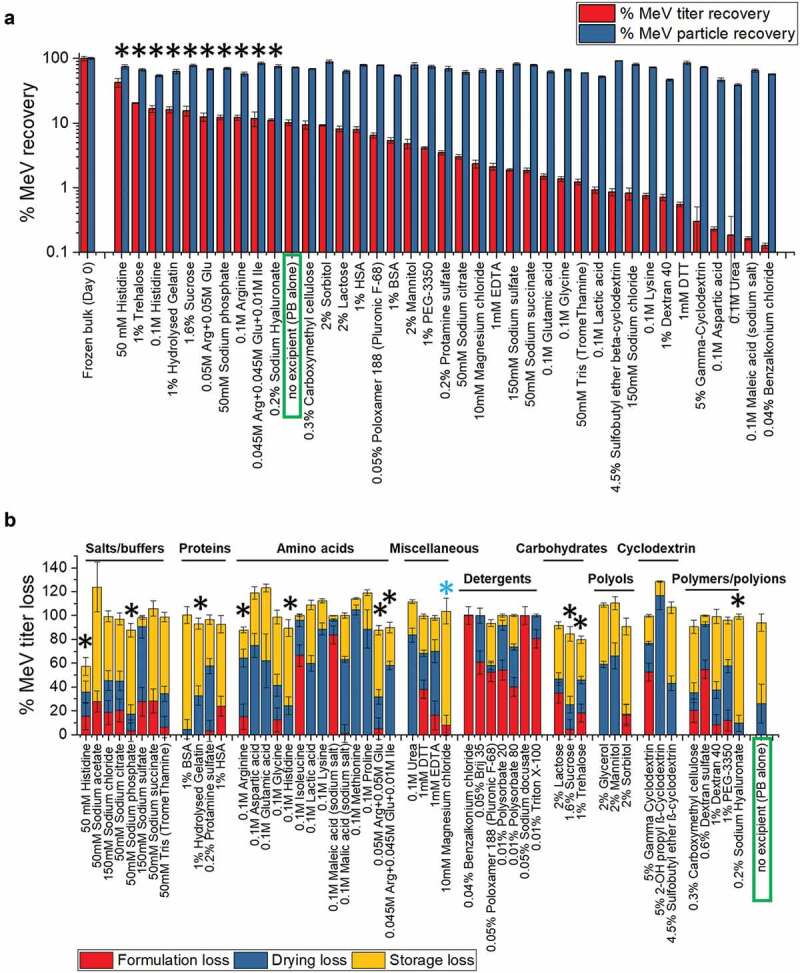
Effect of excipients on stability of Me after drying and storage of MeRu samples for 2 weeks at 25°C. (A) Relative Me viral titers and Me genomes after drying onto LCP discs and storage. Each value is relative to a control sample (frozen liquid bulk stored at −80°C). Error bars represent one standard deviation of quadruplicate experiments. The stabilizing “hits”, in which the infectious viral titer were higher than control (boxed in blue), were marked by black asterisks. (B) Relative Me viral titers losses during formulation, drying and storage for 2 weeks at 25°C in the dried state. Each Me value is relative to a control Me sample (frozen liquid bulk stored at −80°C). An additional excipient, magnesium chloride, which showed stabilizing effect on drying for Me, is marked by blue asterisk. MeRu samples were prepared with 51 individual excipients in a 10 mM phosphate, pH 7.2 buffer at a target titer of 10^2.02^ CCID_50_/disc for each virus. Me viral infectious titers were measured by duplex infectivity RT-qPCR assay, and total viral genome content were determined by genomic RT-qPCR assay. Error bars were calculated via the propagated error of standard deviations from quadruplicate experiments

**Figure 4.**
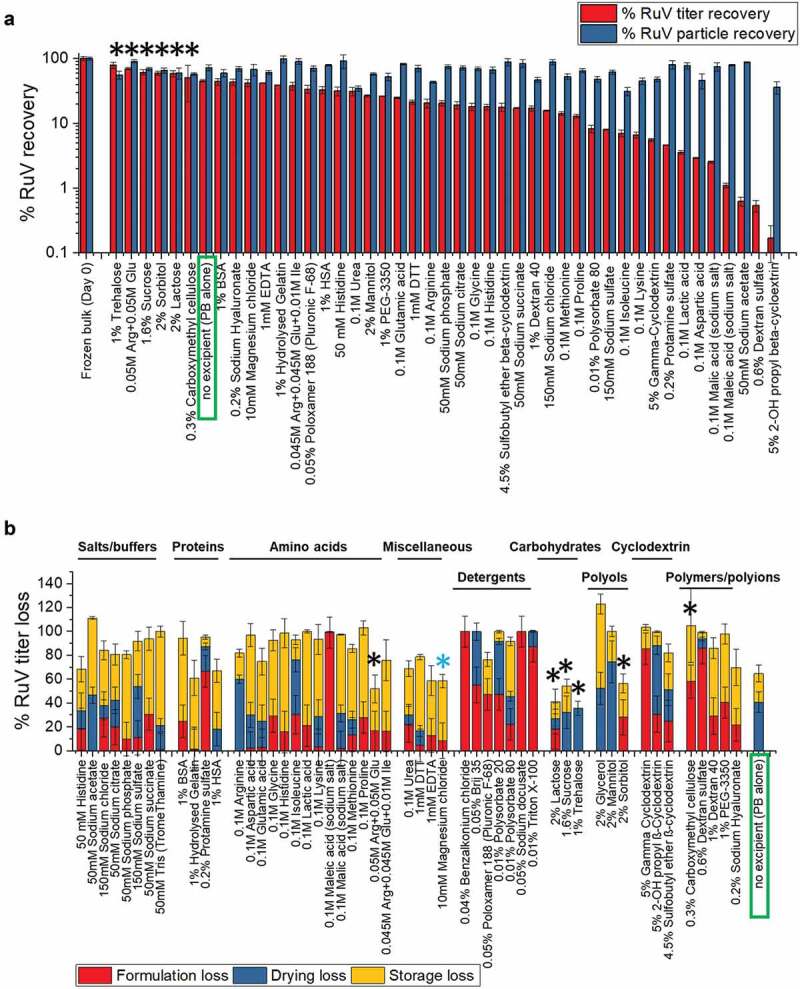
Effect of excipients on stability of Ru after drying and storage of MeRu samples for 2 weeks at 25°C. (A) Relative Ru viral titers and Ru genomes after drying onto LCP discs and storage. Each Ru value is relative to a control Ru sample (frozen liquid bulk stored at −80°C). Error bars represent one standard deviation of quadruplicate experiments. The stabilizing “hits”, in which the infectious viral titer were higher than control (boxed in blue), were marked by black asterisks. (B) Relative Ru viral titers losses during formulation, drying and storage for 2 weeks at 25°C in the dried state. Each Ru value is relative to a control Ru sample (frozen liquid bulk stored at −80°C). An additional excipient, magnesium chloride, which showed stabilizing effect on drying for Ru, is marked by blue asterisk. MeRu samples were prepared with 51 individual excipients in a 10 mM phosphate, pH 7.2 buffer at a target titer of 10^2.02^ CCID_50_/disc for each virus. Ru viral infectious titers were measured by duplex infectivity RT-qPCR assay, and total viral genome content were determined by genomic RT-qPCR assay. Error bars were calculated via the propagated error of standard deviations from quadruplicate experiments

Different excipient categories and types could stabilize MeRu by different mechanisms upon drying onto the MAPs and/or during storage in the solid state. To identify as many potential stabilizers as possible, 51 different excipients, which not only cover diverse excipient categories but are also found on the FDA inactive ingredient guide for parenteral products (with a few exceptions),^51^ were evaluated for their effect on Me and Ru stability as outlined in Figure 2. The excipient concentrations evaluated were selected to ensure efficient evaporative drying onto the LCP discs (i.e., the total weight of excipients that can be dried onto a MAP is limited by the manufacturing process; see discussion). As shown in Figures 3A and 4A for Me and Ru, respectively, the control sample without excipients showed ~10% and ~45% of the infectious virus titer remaining while ~100% total virus particles were recovered after drying and storage. The stabilizing excipient “hits”, 10 for Me and 6 for Ru, in which the infectious viral titer values were higher than control (boxed in green), are marked by black asterisk in Figures 3A and 4A. Among them, three conditions (one arginine mixture and two carbohydrates) were common stabilizers for both Me and Ru. The ability of the additives to differentially stabilize or destabilize Me and Ru titers under these conditions is clearly demonstrated. At the same time, essentially no loss of viral particles across the additives was observed showing that the loss of Me or Ru infectious viral titer was not due to physical loss of viral particles (Figure 3A and 4A).

To better understand the stabilizing and destabilizing effect of each of the individual excipients, we measured the viral infectivity titer of each MeRu sample after formulation (before drying), immediately after drying, and then after storage in the dried state. We then plotted the results by category of excipients as shown in Figures 3B and 4B. Some categories of excipients destabilized these two enveloped viruses upon formulation, for example, detergents which likely disrupted the viral envelope. Thirteen individual “hits” (marked by black * in Figures 3B and 4B) stabilized Me and/or Ru during drying and/or storage to different extents. Interestingly, magnesium chloride showed a stabilizing effect on drying for both Me and Ru (marked by blue * in Figures 3B and Figure 4B), and thus was included as an additional “hit” for further evaluation. In summary, 14 additives across various classes of excipients maintained equivalent or higher infectious viral titers after drying and storage for both Me or Ru (compared to no excipient control) and were identified as “hits”. These included amino acids and salts (histidine, magnesium chloride, sodium phosphate arginine and arginine mixtures), sugars and sugar alcohols (trehalose, sucrose, sorbitol, lactose), and polymers (hydrolyzed gelatin, sodium hyaluronate, carboxymethyl cellulose).

To further evaluate these 14 “hits” and to determine their optimal effective concentrations, each stabilizer was titrated, from 0.5 to 1.5-fold range of concentration used in the initial screen, for their stabilizing effect on Me and Ru during drying and storage at 25°C, 2 weeks (data not shown). Based on these results, 50 mM histidine, 0.1 M arginine, 1% hydrolyzed gelatin, and 1% trehalose looked promising, with the latter two (hydrolyzed gelatin and trehalose) as common stabilizers for both Me and Ru. Sodium hyaluronate and carboxymethyl cellulose were stabilizing for Ru, but were not further evaluated due to high solution viscosity and cost. Three other additives (25 mM phosphate, 1% sorbitol, and 5 mM magnesium chloride) showed less consistent benefit, however, were nonetheless further evaluated to assess potential synergistic/additive effects with other stabilizers. Finally, the use of phosphate buffer (with and without NaCl) across the pH range of 6.0 to 7.4 was evaluated. NaCl addition showed no improvement in Me or Ru stability and pH 6.8 was found to be the best solution pH to retain Me and Ru infectious viral titers under these conditions (data not shown).

**Figure 5.**
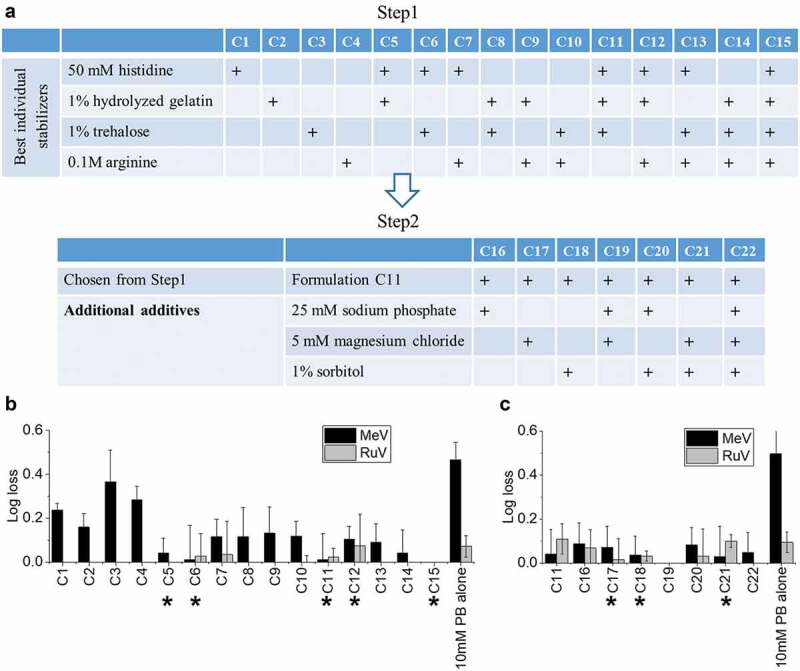
Effect of combining excipients to further improve the stability of MeRu during drying and storage in dried state on LCP discs. (A) A two-step excipient combination screening strategy was used (Steps 1 and 2), (B) Me and Ru viral infectivity titer log loss during Step 1 screening, and (C) Me and Ru viral infectivity titer log loss during Step 2 screening. Log loss values are relative to a control sample (frozen liquid bulk stored at −80°C). MeRu was formulated with different combinations of excipients in 10 mM phosphate buffer, pH 6.8, dried onto LCP discs at a target titer of 10^2.02^ CCID_50_/disc for each virus, stored for two weeks at 25°C in the dried state, and viral titers were measured by infectivity RT-qPCR assay. Error bars represent the propagated error of standard deviations from three replicate runs (quadruplicate experiments for each rep run). (*) indicates MeRu candidate formulations selected for further evaluation in storage stability studies (see Figure 6–8)

Due to the large number of possible combination formulations using the top seven “hits”, the combination screen study was divided into two steps as shown in Figure 5. For Step 1, we screened combinations containing the four top individual stabilizers in various combinations (Formulations C1-C15). For Step 2, we screened additional excipients with one of the best combination formulations from Step 1 (Formulation C11). As seen in Figure 5B for Step 1, individual excipients stabilized Me and Ru (0.2–0.4 log_10_ loss for C1 – C4), and combination formulations showed trend of improved stability (<0.2 log_10_ loss observed for C5 – C15). Formulations C5, C6, C11, C12, and C15 were identified as promising stabilizing combination formulations for Me. Although both formulations C11 and C15 showed the most stabilizing effects, C15 contained arginine, an excipient appearing to be a suboptimal component in other formulations, thus, formulation C11 was selected for further evaluation. For Step 2, as shown in Figure 5C, both Me and Ru showed good stability (< 0.2 log_10_ loss) in all of the combination formulations (C11, C16 thru-C22) compared to buffer alone control. The five most stabilizing formulations as ranked by average Me viral titer remaining from Step 1, along with three additional formulations from Step 2 (which include the additional excipients Mg, sorbitol, or both) were selected for real-time/accelerated stability studies (marked by * in Figure 5B and C). The stabilizing effects of eight selected candidate formulations on Me and Ru were also confirmed with CCID_50_ assays (data not shown).

**Figure 6.**
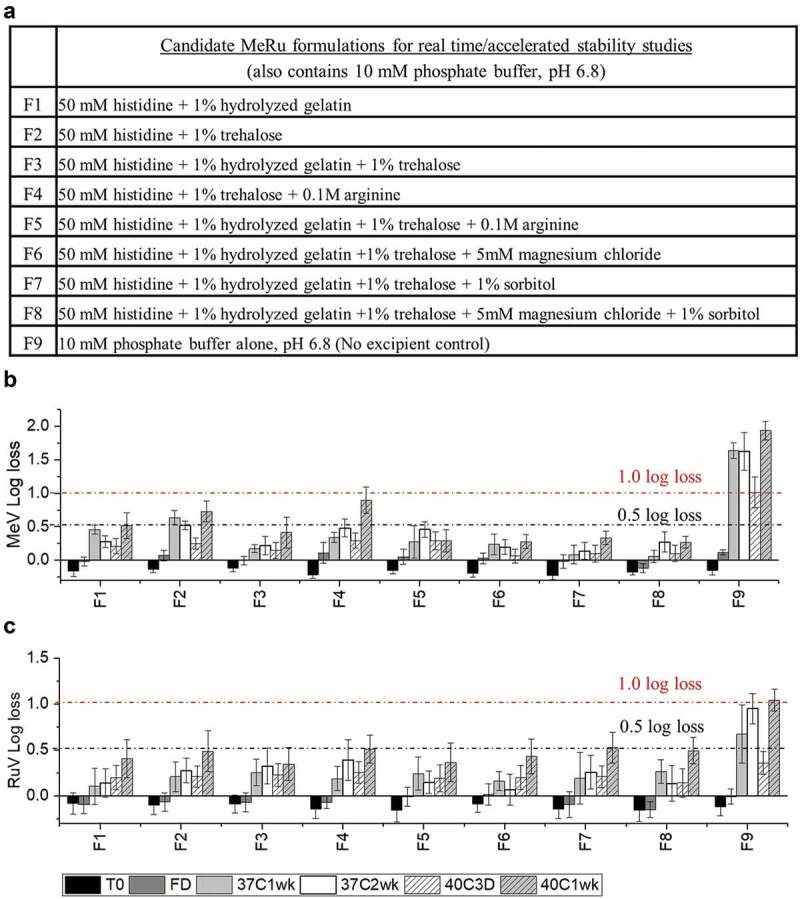
Thermal stability of MeRu in candidate formulations at stressed temperatures of 37°C and 40°C. (A) Composition of candidate MeRu candidate formulations at a target titer of 10^2.02^ CCID_50_/disc for each virus, (B) Me viral infectivity titer log loss, and (C) Ru viral infectivity titer log loss. Log_10_ loss is shown relative to values of control sample (frozen liquid bulk stored at −80°C). Each candidate MeRu formulation was prepared (T0), dried onto LCP discs (fresh dried, FD), stored for 1 or 2 weeks at 37°C, or 3 and 7 days at 40°C in the dried state, and assayed by infectivity RT-qPCR. Log loss values are relative to control sample (frozen liquid bulk stored at −80°C). Error bars were calculated via the propagated error of standard deviations from quadruplicate experiments

**Figure 7.**
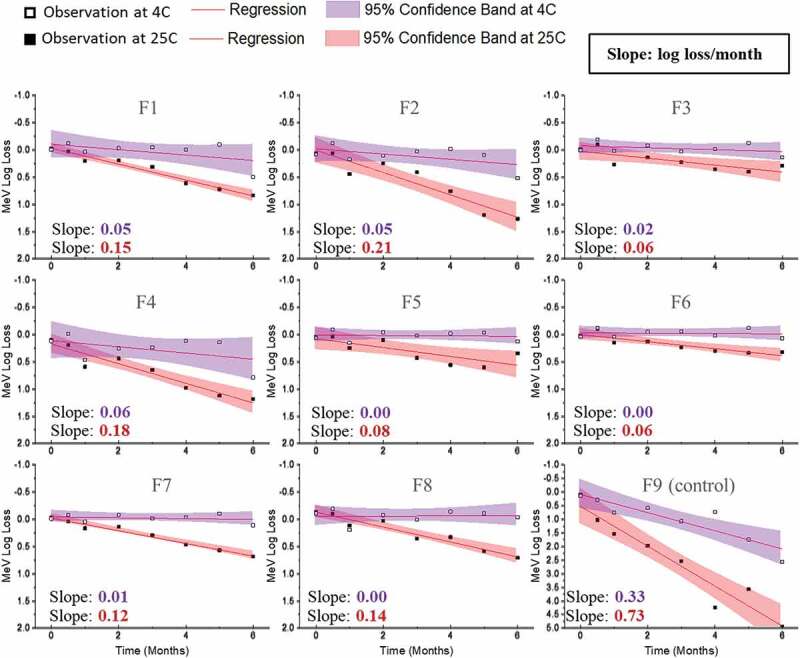
Stability profile of Me during accelerated (25°C) and real-time (2–8°C) stability studies with candidate MeRu formulations. Each candidate MeRu formulation was prepared, dried and then stored for 0.5, 1, 2, 3, 4, 5, and 6 months in the dried state. The *in vitro* potency of Me was measured by an infectivity RT-qPCR assay. Log_10_ loss values are relative to a control Me sample (frozen liquid bulk stored at −80°C). Solid lines (log loss/month) represent regression loss of Me viral titers (vs. control) and shaded bands represent 95% CI bands. Each titer was the average of quadruplicate measurements. See Figure 6 for composition of candidate MeRu formulations F1-F9

**Figure 8.**
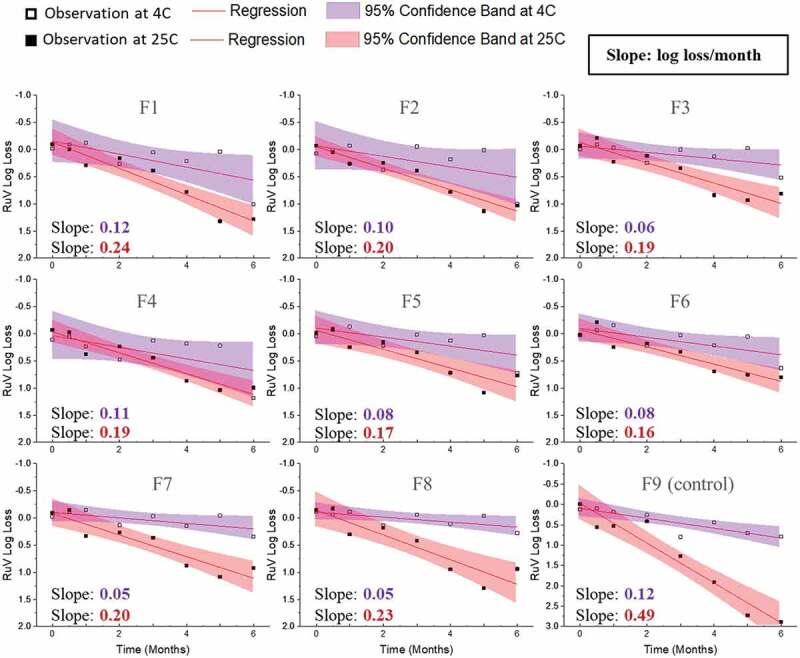
Stability profile of Ru during accelerated (25°C) and real-time (2–8°C) stability studies with candidate MeRu formulations. Each candidate MeRu formulation was prepared, dried and then stored for 0.5, 1, 2, 3, 4, 5, and 6 months in the dried state. The *in vitro* potency of Ru was measured by an infectivity RT-qPCR assay. Log loss values are relative to a control Ru sample (frozen liquid bulk stored at −80°C). Solid lines (log loss/month) represent regression loss of Ru viral titers (vs. control) and shaded bands represent 95% CI bands. Each titer was the average of quadruplicate measurements. See Figure 6 for composition of candidate MeRu formulations F1-F9

### Accelerated and real-time stability studies with candidate MeRu formulations in the dried state

The selected eight candidate MeRu formulations are listed in Figure 6A (Formulations F1 – F8) along with a no excipient control (Formulation F9). First, we evaluated the formulations under conditions of a target thermal stability test for live vaccines from the World Health Organization (<1 log_10_ loss after 1 week storage at 37°C).^12^ In addition, MeRu stability in the dried state in these 8 candidate formulations was examined at different timepoints at both 37°C and 40°C. As shown in Figure 6B and 6C, all eight candidate formulations met the target thermal stability test for both the Me and Ru components, respectively. Across the various timepoints and formulations, Me samples containing histidine, hydrolyzed gelatin, trehalose (F3, F5, F6, F7, and F8) showed lowest potency losses (<0.5 log_10_ loss in all tested conditions) while Ru was similarly stable in all candidate formulations (<0.5 log_10_ loss in all tested conditions).

A 6-month accelerated (25°C) and real-time (4°C) stability study was then performed to evaluate Me and Ru stability profiles in the 8 candidate MeRu formulations in the dried state on LCP discs. To better account for assay variability, a control for each virus (frozen liquid bulk sample stored at −80°C) was run in the assay for each stability timepoint, and the log loss of the viral infectious titer was calculated vs. the control. The slope of the titer log loss over storage time and 95% confidence interval were then calculated,^52^ using linear regression fit curves (i.e., smaller slope value indicated higher stability), and results were compared. As shown in Figure 7, Me was overall stable over 6 months at 4°C with 0.0–0.4 log_10_ loss observed in all tested conditions (calculated from the slope of linear regression). At 25°C for 6 months, 0.4 to 1.3 log_10_ loss of titer was observed across the eight candidate formulations. In contrast, the control formulation (F9) showed much greater losses of Me viral titers under these conditions. Similar stability data plots are shown in Figure 8 for Ru. Ru was more stable in candidate formulations (F3, F5, F6, F7, and F8), with 0.3–0.5 log_10_ loss observed at 4°C. Somewhat surprisingly, based on the 6 month 4°C stability data, Me was relatively more stable than rubella in the candidate formulations.

To further confirm these results, CCID_50_ based viral titer assays and genomic RT-qPCR assays were also performed with the same stability samples on selected samples (0-, 1-, 3-, or 6-month storage at 4°C, or 25°C in the dried state). As shown in Supplemental Figures S2 (4°C) and S3 (25°C), infectivity RT-qPCR and CCID_50_ stability results for the eight candidate formulations were in overall good agreement in measuring Me or Ru titer log loss across the various timepoints and temperatures. Genomic RT-qPCR assays were also performed to measure the total viral particle content in the same stability samples. As shown in Supplemental Figures S4 (4°C) and S5 (25°C), essentially no losses of total Me or Ru viral particles were observed across the candidate formulations indicating the loss of virus titer during storage was not due to physical loss of viral particles. Interestingly, for the control F9 formulation (no excipients), a much greater loss of viral titers and a small yet observable physical loss of viral particles was observed for both Me and Ru components.

## Discussion

**Figure 9.**
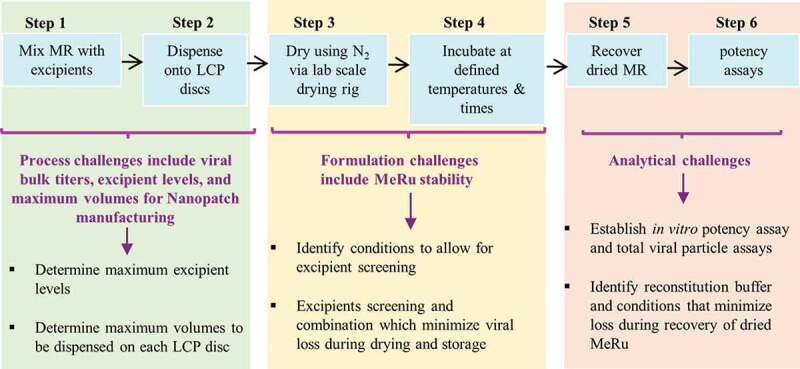
Formulation development challenges to develop a dried, live-attenuated MeRu combination vaccine for use in the Nanopatch^TM^ delivery system. Steps 1, 2 cover processing challenges using a scale-down lab model of Nanopatch^TM^ coating/drying, Steps 3, 4 show virus stability challenges, and Steps 5, 6 summarize analytical testing challenges encountered during formulation development

We recently demonstrated that a trivalent inactivated polio (tIPV) vaccine can be formulated and stabilized by a combination of excipients for use with Nanopatch™ microneedles as evaluated using a scale-down, laboratory model of the coating/drying process.^31^ In addition, an inactivated influenza virus vaccine was successfully formulated for use with Nanopatch™.^13^ However, compared to these inactivated viral vaccines (which are currently formulated as simple, liquid dosage forms as commercial products), live attenuated enveloped viruses such as Me and Ru are inherently less stable and are formulated as lyophilized commercial products containing numerous, complex mixtures of excipients to obtain adequate storage stability.^53^ Moreover, it is more difficult to measure the stability profile of live viral vaccines using *in vitro* potency assays measuring viral replication (instead of the ability of an inactivated viral vaccine to bind an antibody reagent). To this end, we had to overcome several technical challenges to formulate live, attenuated enveloped Me and Ru viruses in the Nanopatch™, as outlined in Figure 9 and discussed in detail below, including (1) process constraints such as maximum virus bulk titers as well as limits to excipient types/levels compatible with the Nanopatch^TM^ manufacturing steps, (2) effect of formulation excipients on the stability of two different viruses during drying and storage in the dried state, and (3) development of appropriate analytical methods and experimental conditions to monitor virus titer yields and stability. As each of these challenges were addressed experimentally, we were then able to identify stable candidate formulations for measles and rubella combination vaccine that can be used with the Nanopatch^TM^ microneedle technology.

As shown in Steps 1 and 2 in Figure 9, the manufacturing processes for the bulk viruses and Nanopatch^TM^ microneedle each have some inherent constraints in terms of this formulation development work. For example, the maximum titers of the viral bulks as well as the weight and volume limitations that can be effectively coated and dried during the Nanopatch™ processing must be considered. The Me and Ru viral bulks used in this work contained various excipients to ensure virus stability during frozen storage (and shipping) and during subsequent freeze-thaw. The excipient levels present in the bulk, however, exceeded the solids content limit for effective coating and drying, and some of the excipients themselves were not effective for virus stabilization for this purpose (data not shown). To overcome these challenges using the laboratory scale-down model that mimics the Nanopatch^TM^ drying/coating process, the Me and Ru viral bulks were each diluted at least 20-fold into candidate formulations with much lower solids content. Since only ≤10 µL of this formulated MeRu solution can be loaded onto each 6 mm disc in a 96-well plate format, this volume limit constrains the amount of virus that can be added. These trade-offs in terms of maximum excipient weight and volume limits led to the requirement of using diluted viral bulks.

Based on these considerations, the Me and Ru viral titer added to each LCP disc was 100 CCID_50_, which translated to ~1/10^th^ of a full human dose per disc. This dose was considered to be sufficiently representative for the formulation development work described herein. To prepare Me and Ru formulations at a full human dose for clinical use, a virus concentration method has been subsequently developed to allow for a full human dose to be coated on the MAPs (communication from Vaxxas Pty Ltd). On the other hand, the intradermal (ID) route of administration has dose sparing potential,^54^ and Nanopatch^TM^ targets vaccine antigens to the epidermal and dermal layers of the skin. Such targeted ID delivery in Nanopatch^TM^ format has shown dose sparing, for instance, as little as 1/40^th^ of a human dose of IPV2 was able to elicit protective levels of neutralizing antibodies from a single dose in animals.^17^ Although the MeRu combination vaccine in the Nanopatch^TM^ format may not require the full human dose that is currently administered (by subcutaneous delivery via needle and syringe^11^), preclinical and clinical evaluations will be required to establish such a dose sparing potential.

As shown in Steps 3 and 4 in Figure 9, the viral stability challenges include identifying stabilizing excipients (at certain weight constraints) for two different live attenuated viruses, Me and Ru. The goal was to identify excipients to minimize Me and Ru viral titer losses during each of the different stages of the process including formulation preparation, drying onto the LCP discs, and storage in the dried state. Commercially available Me and Ru containing vaccines are lyophilized in glass vials, stored at 2–8°C, and require reconstitution with a sterile diluent prior to subcutaneous administration by a needle/syringe.^53^ In contrast, the Me and Ru viruses being formulated for use in the Nanopatch^TM^ technology will be exposed to different stresses, and thus may require a different combination of excipients to stabilize them. For example, the lyophilization process is initiated by freezing followed by removal of bulk water by the sublimation of ice.^55^ In contrast, there is no freezing step involved in this Nanopatch drying process, but rather it involves rapid evaporative drying (of very small volumes, e.g., multiple dispensing of pL volumes onto a MAP) to remove the bulk water from the liquid state under ambient conditions. Other processing differences for lyophilized vs MAP formulations of a Me and Re combination vaccine include contact surfaces (glass vials vs. LCP discs) and the reconstitution/administration procedures (saline diluent to reconstitute followed by needle/syringe vs. reconstitution *in vivo*; see below).

The top candidate formulations identified in this work to stabilize Me and Ru during evaporative drying and subsequent storage included specific types and classes of excipients including histidine, phosphate, hydrolyzed gelatin, magnesium chloride, trehalose, and sorbitol. Since live viral vaccine formulations typically require a complex mixture of excipients to stabilize viruses, the mechanisms of excipient stabilization (or destabilization) are often not well-understood, and thus stabilizers are selected by trial-and-error empirical screenings.^56,57^ Destabilizers such as surfactants likely disrupt the viral lipid envelope of Me and Ru. In addition, surfactants did not perform well during the drying process (i.e., formed a thin layer that easily detached from the LCP surface). For Me and Ru virus stabilizers identified in this work, histidine and phosphate are commonly used buffering agents to control the solution pH.^58^ As an amino acid, histidine can also act as an free radical scavenger,^58^ and may also stabilize viral proteins via non-covalent histidine–protein interactions in the solid state.^59^ Hydrolyzed gelatin is a protein-based stabilizer used in many live viral vaccine formulations and is probably effective due to its interaction with viral particles as well as its ability to inhibit surface adsorption.^56,58^ During drying, trehalose and sorbitol may substitute for the water-layer that surrounds the viral coat proteins and lipid membrane, by providing stabilizing H-bonds, thus diminishing structural alternations of protein and lipid assemblies caused by dehydration.^56,58^ Mg^2+^ was also identified as a virus stabilizer in this study, and it has been reported previously as a Me stabilizer during spray drying.^60^ Possible mechanisms of Mg^2+^ – induced virus stabilization include direct binding to viral proteins and/or effects on viral protein’s conformational flexibility.^58^

While sugars and polyols are generally used as either cryoprotectants or lyoprotectants to stabilize biological drugs and vaccines during lyophilization (at ~10-15% by weight concentrations), only lower concentrations (~1-2%) are compatible with nitrogen-flow evaporation used in this work. Interestingly, magnesium chloride and sorbitol appeared to mitigate viral titer losses during drying, while histidine, arginine, and carbohydrates appeared to mitigate storage losses in a dried state. When combined, the Me and Ru viral titer losses during drying and during accelerated storage conditions in the dried state (e.g., candidate formulations F3, F6, and F8) showed improved stability, suggesting additive/synergistic effects of these excipients. Each of the candidate formulations contained less than 3% total solid contents (w/v). In a different microneedle system for Me, Edens *et al* reported that with higher amount of excipients (7.5% trehalose, 150 mM threonine, and 1% CMC), and a double coating process (the microneedles were first dipped into Me solutions with excipients and dried by vacuum drying, and then the Me-coated microneedles were dipped into a sugar – polyvinyl alcohol matrix solution and dried again by lyophilization), Me retained 100% *in vitro* potency at 4 and 25°C for up to 4 months.^21^ Such a formulation contains ~4-fold higher levels of excipients than the candidate Me Ru formulations reported in this study, and such elevated excipient levels are not compatible with the laboratory scale-down model of Nanopatch^TM^ drying process (i.e., drying could not be accomplished; data not shown). Based on the lead excipients identified in this work, future work on excipient concentration optimization to finalize a manufacturing process can be performed using a design of experiment approach to define the formulation design space. This approach is consistent with other work from our laboratory with lyophilization of live virus vaccine candidates using a formulation strategy that employs both semi-empirical screening of excipients (to identify hits and reduce the number of variables) followed by formulation process optimization using design of experiments (DOE) experiments.^61^

Finally, as shown in Steps 5 and 6 in Figure 9, identifying sensitive analytical methods and experimental conditions to monitor Me and Ru virus titers was a critical part of this formulation development work. This challenge included first identifying an appropriate rehydration solution to rehydrate and recover the MeRu viruses from the LCP discs. To this end, it was found the addition of a blocking protein such as bovine serum albumin was required along with a specific sequence of mixing (see methods). Second, an assay was needed with the ability to monitor Me and Ru viral titers at low viral input levels (≤100 CCID_50_) during drying and after additional titer losses during storage in the dried state. In addition, a large number of samples containing individual excipients and their combinations needed to be evaluated for their ability to stabilize Me and Ru.

To this end, viral titers (infectivity RT-qPCR) and viral genomes (genome RT-qPCR) assays were utilized. These RT-qPCR methods displayed several key advantages for formulation development work over the traditional viral cytopathic effect (CPE) assays such as CCID_50_. The RT-qPCR assays enabled testing of both Me and Ru viruses in one assay, abundant sample throughput (96-well format), and assay flexibility (assay plates could be stored at −80°C until RT-qPCR analysis was performed). The RT-qPCR assay showed very good correlations with the traditional CCID_50_ assays in terms of monitoring virus stability (Figure 1). In addition, the RT-qPCR method and was more sensitive than CCID50 assay for detecting low viral inputs (upon optimization with proper infection method and primer/probe sets, Supplemental Fig. S1), and thus the RT-qPCR assay was ideal for formulation development.

For the Me and Ru stability assessments described in this work, a statistical analysis using linear regression was used to compare the relative stability profiles of Me and Ru in the 8 candidate formulations.^52^ There are many advantages to this approach including determining viral stability profiles using all the stability data over 6 months and thus being less affected by individual timepoints and potential outliers. In addition, the candidate formulations can be easily rank ordered by determining the slopes of linear regression of viral titer loss per month. The Me and Ru viruses in the eight candidate formulations met a target thermal stability test for live vaccines (<1 log_10_ loss after 1 week storage at 37°C) from the World Health Organization.^52^ By evaluating virus stability over a six-month real-time and accelerated stability study, the top three stabilizing candidate formulations were identified as formulations F3, F6, and F8 (rank ordered by the slopes of linear regression). In the case of candidate formulation F6, the log_10_ loss of viral titers (vs. a frozen control sample) after 6 months at 2–8°C was < 0.1 for Me and ~0.4 for Ru. After storage for 6 months at 25°C, the log_10_ loss of viral titers (vs. a frozen control sample) after was 0.5 and 1.0 for Me and Ru, respectively.

In terms of ongoing and future work, LCP discs were used as surrogate for LCP-based microprojections to enable much faster experimental throughput at a laboratory scale. The ability of this scale-down model to translate into a viable manufacturing process needs to be considered and could potentially be a limitation. First, the different drying kinetics is a possible difference that must be evaluated in terms of virus yields and storage stability when the candidate formulations are dispensed onto MAPs in a manufacturing setting. In general, the longer drying times required of 10 µL on LCP disc (lab-scale) can be considered harsher treatment than the rapid drying of pL volumes placed on individual projections on MAP (manufacturing scale). Such work is ongoing at Vaxxas Pty Ltd allowing viral titers in the presence of promising excipients to be evaluated on MAPs under manufacturing conditions. Second, the effect of varying residual moisture levels remaining after drying on virus yields and stability needs to be further assessed. Due to low excipient levels, moisture measurements are experimentally challenging, but preliminary data indicate with 10 µL of a candidate formulation dried onto a single LCP-disc, there was between ~20-40 µg water with ~300 µg excipient (data not shown). Finally, the resultant morphology of the coating once dried onto the MAP projections is an additional selection criterion for selection of a final formulation. To evaluate the morphology and vaccine delivery, variety of methods have been established in Vaxxas Pty Ltd including scanning electron microscopy (SEM),^26^ contact angles, membrane transfer assay, and porcine skin delivery assay.^14^ The candidate formulations were routinely evaluated through SEM and membrane transfer assay at Vaxxas Pty Ltd to down-select formulations that are likely to penetrate skin and deliver the payload efficiently.

## Conclusions

In this work, we demonstrate the feasibility of formulating and stabilizing a live attenuated Me and Ru combination vaccine in the Nanopatch^TM^ microneedle system during processing and storage. A combination of high-throughput, RT-qPCR-based assays (to monitor the *in vitro* stability of both live attenuated viruses) and a scaled-down, custom-built evaporative drying system (to mimic the Nanopatch^TM^ vaccine coating process) were used to enable extensive formulation development work. A series of excipient screening and real-time and accelerated stability studies demonstrated that MeRu could be stabilized during the Nanopatch^TM^ drying process and during storage in the dried state. Several promising candidate formulations for a live, attenuated Me and Ru combination viral vaccine for use with Nanopatch^TM^ were identified. Top three candidate formulations (e.g., F3, F6, and F8) resulted in greatly improved storage stability for Me and Ru over 6 months at 2–8°C, and key stability results were confirmed by the more commonly used CCID_50_ viral infectivity assays. Based on these encouraging results, both preclinical and process development studies are in progress with Me and Ru in these candidate formulations with goal to initiate clinical feasibility of Nanopatch^TM^ microneedle-based dosage forms containing live, attenuated viral vaccines for use in LMICs.
